# Exactech Opteon Femoral Component Fracture 12 Years after Arthroplasty

**DOI:** 10.1155/2016/1789197

**Published:** 2016-02-03

**Authors:** Shaun P. Patel, Valentin Antoci, John J. Kadzielski, Mark S. Vrahas

**Affiliations:** Department of Orthopaedic Surgery, Massachusetts General Hospital, Boston, MA 02114, USA

## Abstract

Arthroplasty implant fracture is a rare but critical complication that requires difficult revision surgery, often with poor results, patient disability, and significant cost. Several reports show component fracture either at the stem or at the neck interface after a relatively short postoperative course. We report such failure after 12 years, suggesting no safe period after which femoral implant fracture does not occur.

## 1. Introduction

Arthroplasty has been ranked in the top 10 list of medical innovations, improving quality of life and prolonging activity into old age [1]. However, complications such as femoral component failure, even though rare, can have significant sequelae, including death. A retrospective survey performed by the American Association of Hip and Knee Surgeons estimated the prevalence of femoral stem fractures to be 0.27% [2].

Previous case reports have documented the failure of femoral neck and stems of JRI-Furlong hydroxyapatite-ceramic coated femoral prostheses (Joint Replacement Instrumentation Limited, London, UK) [3]. Failures occurred in patients with a BMI over 25 and were attributed to fatigue secondary to high stress over time. Another case report series described failures of the Omnifit cobalt chrome femoral component (Osteonics, Allendale, NJ) occurring in the femoral neck adjacent to the modular head. Macroscopic and microstructural analysis revealed that the cause of failure had been initiated by intergranular corrosion, crack growth through fatigue, and resultant failure from tensile stress. An annealing step that would prevent intergranular carbide precipitation could prevent such failure and would represent a viable solution to repeat occurrence [4].

We now report on the failure of the Exactech Opteon femoral component (Exactech, Gainesville, FL) that may have subsequently led to death. A previous case report series documented failure of these components at an average of 43 months, with early fatigue and subsequent fracture thought to be resulting from laser etching and marking of the metal [5]. Interestingly, unlike previously reported, the component failure we observed occurred much later at 12 years after implantation.

## 2. Case Presentation

**Figure 1:**
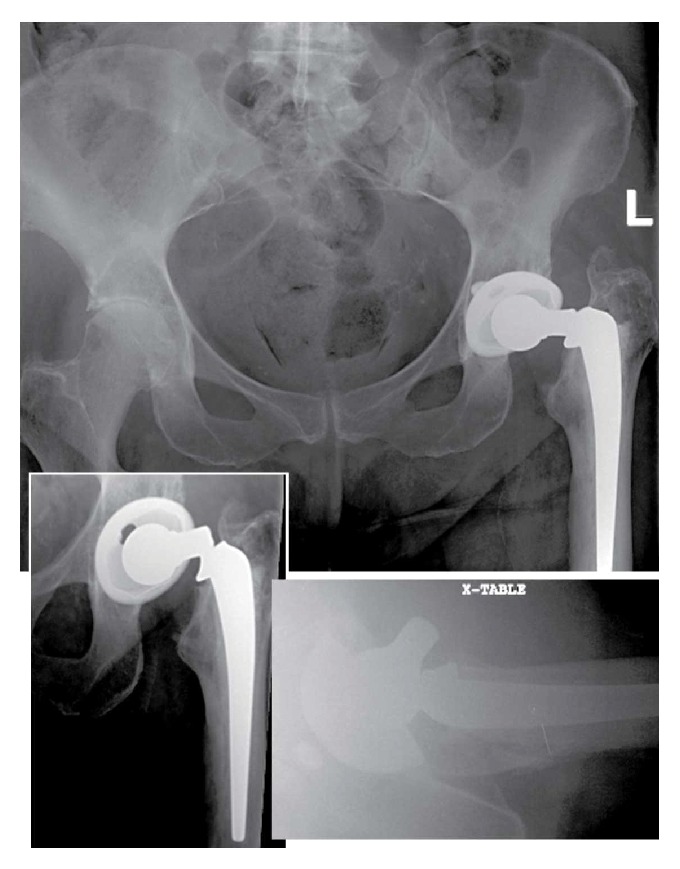
Admission radiographs show femoral component fracture at the neck-shoulder junction at 12 years after implantation.

An 82-year-old female, with a BMI of 33.9, was transferred to our hospital from an outside facility with a diagnosis of a failed left hip prosthesis. The implant had fractured in the area of the femoral neck adjacent to the modular head (Figure 1).

The patient had undergone left total hip arthroplasty twelve years prior to presentation for osteoarthritis. The femoral component was a cemented Exactech Opteon stem, size 2, with a 28 mm, +5 mm femoral head. The acetabular component was a 54 mm Duraloc cup with an Enduron polyethylene liner (DePuy Inc., Warsaw, IN). The patient had a total knee arthroplasty performed in the ipsilateral extremity three years prior to the total hip arthroplasty also for osteoarthritis. Three months after the total hip arthroplasty, the patient developed a* Staphylococcus epidermidis* periprosthetic infection in her knee and underwent exchange arthroplasty later that same year. Only the total knee implant had been affected and revised, whereas the total hip implant was unaffected. The patient subsequently underwent successful rehabilitation and had been an independent community ambulator thereafter.

Upon presentation, the patient denied shortness of breath, chest pain, fevers, chills, night sweats, or recent travel. Five month prior to presentation, the patient had dental work with appropriate antibiotic prophylaxis. The critical event most likely occurred several days prior to presentation when the patient slipped and fell in the bathroom. Plain radiographs revealed a fracture of the femoral component at the area of the femoral neck adjacent to the modular head (Figure 1). The stem appeared well seated with no evidence of infection, loosening, malalignment, or periprosthetic fracture. Examination and imaging of the ipsilateral knee were unremarkable.

Surgery was subsequently planned for component retrieval and revision arthroplasty. Under general anesthesia, the patient was placed in the lateral decubitus position. The previous incision was opened, the subcutaneous tissues were dissected, and the plane beneath the tensor fascia lata was elevated. The sciatic nerve was identified and noted to be in its normal position. The short external rotators were scarred and adhered to the joint capsule. This layer was dissected as a full thickness flap. The quadratus femoris muscle appeared healthy distally. The capsule was then incised, revealing a small amount of serous fluid within the joint. The posterior capsule was removed and the remainder of the anterior rim of the cup was exposed. Tissue samples were sent for frozen section.

**Figure 2:**
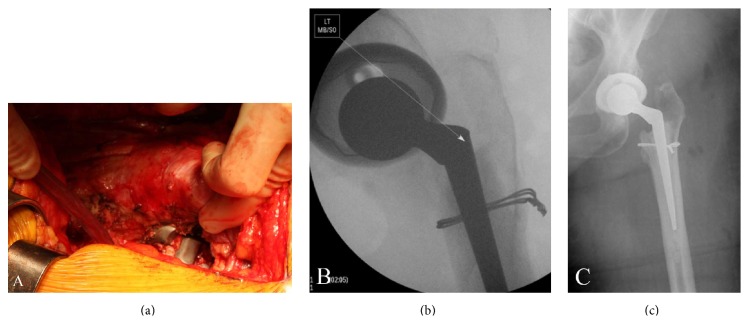
(a) Intraoperative femoral component fracture and separation. (b) A new locking ring and liner were placed in the preexisting well-fixed Duraloc cup. (c) Postoperative imaging confirms stable implant positioning.

The top of the fractured femoral stem was then located (Figure 2(a)). The soft tissues were removed from the area of the greater trochanter and the cement around the posterior shoulder and around the collar of the prosthesis was removed. A carbide punch was used to drive the prosthesis out of the cement mantle. Examination of the cement mantle after removal of the prosthesis showed a small crack proximally which was suspected to proceed into the calcar. To stabilize this crack, a 16-gauge doubled wire was passed around the femur, proximal to the lesser trochanter, and tightened into place (Figure 2(b)).

The remainder of the capsule was removed around the rim of the acetabulum. There was some suspicion for an infection based on frozen sections sent earlier and tissue cultures were therefore taken. Once the rim of the cup had been exposed, a drill hole was placed into the cup and a screw was used to assist with removal of the polyethylene liner. As documented previously, this was a Duraloc cup. The old locking ring was removed and the acetabulum was then irrigated with bacitracin solution. A new locking ring was placed in the well-fixed Duraloc cup and a new polyethylene liner was placed. A Duraloc Marathon cup acetabular liner was used with a 32 mm inner diameter and a 54 mm outer diameter with a neutral face (Figure 2(b)).

The femoral canal was irrigated and dried. The cement mantle was intact. A size 9 Heritage stem was then cemented in place. Once the cement was hardened, the hip was trialed. The hip pistoned in an unacceptable amount even with a 10.5 mm neck length with a 32 mm head. Cement was therefore removed from around the top of the prosthesis and the prosthesis was driven out of the cement mantle without difficulty. Cement was then again placed within the previous cement mantle and the prosthesis was cemented one centimeter higher than the previous attempt. With the cement hardened, the prosthesis was trialed again and the hip was stable with a +7 mm neck (Figure 2(c)).

**Figure 3:**
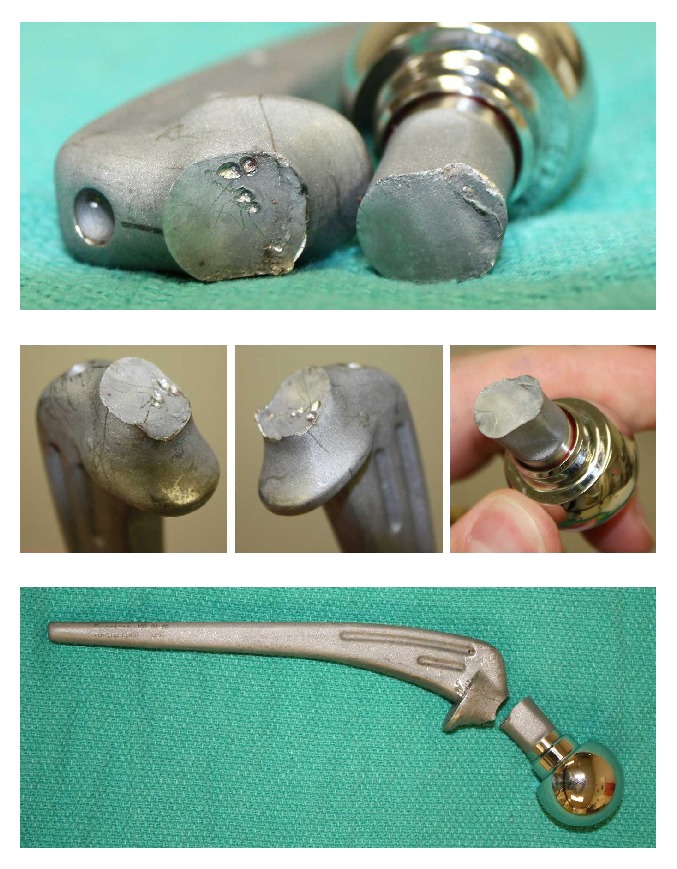
Implant examination from different perspectives shows no gross mechanical or chemical degradation at the fracture site.

The wound was then copiously irrigated. The quadratus femoris and short external rotators were reapproximated to the greater trochanter along with some capsule to provide hip stability. The fascia lata was closed and the remainder of the wound was closed in layers. The implant was examined closely for signs of mechanical or chemical wear (Figure 3). No specific factor was identified in the femoral stem fracture. Intraoperative cultures ultimately returned negative.

## 3. Discussion

Early analysis of femoral component failure after total hip arthroplasty cited a number of possible causes including design configuration, improper metallic alloy, quality of cement, surgical technique, undersizing of prosthesis, and patient characteristics [6–9]. Intergranular corrosion has been shown to cause failure in cobalt alloy femoral stems in two different case report series [4, 10]. Implant handling during implantation is also important and may result in notches or other material defects that can weaken the implant structure and ultimately lead to failure. In our current case, we did not observe any specific notching, although we cannot confirm that such a defect was not present before the implant fracture.

As the obesity epidemic propagates through the arthroplasty population, it raises the question of mechanical overload of implant materials and their long term survival, particularly in conjunction with smaller size implants which may be necessary in these patients. Although the American Academy of Orthopaedic Surgery (AAOS) recommends a BMI cut-off of 40, this is still a morbidly obese population and such a body habitus may overly stress the smaller size implants and lead to implant fracture. One such case report series noted that failure of femoral components occurred in patients with a BMI greater than normal [3], while another study found no significant difference in the complication rate between obese and nonobese patients [11].

In addition to increased BMI as a potential risk factor for implant failure, implant and material analysis studies have demonstrated decreased failure loads with increased loading cycles. In particular, Bergmann et al. have even shown that peak forces generated while stumbling, as occurred in this case, can be nearly three times as high as those generated by walking [12].

Implant design and postmanufacturing processing are also crucial factors that determine the success of the implant. In a six-year period at one institution, 113 primary and revision total hip arthroplasties were performed using the Exactech Opteon femoral component. Two cases were found to have undergone early fatigue fractures secondary to laser etching at the neck-shoulder junction. Similarly, another case report series also noted failure of femoral components associated with laser etching of Precoat femoral component prostheses (Precoat and Precoat Plus; Zimmer, Warsaw, IN) [9]. The Exactech manufacturer had recalled all stems that had undergone laser etching in this area and no other reports of implant failure have since surfaced [5]. We have closely analyzed the implant and fracture surfaces in this report and the failure seems to be similarly related to the laser etching at the interface, which, in turn, created a stress riser and fatigue fracture. It is important to note, however, that patients in previous studies showed failure much earlier, 43 months on average, while we currently report on the possibility of fracture much later, even after 12 years of normal use.

In this case report, we show a unique circumstance of implant failure over 12 years after implantation. The primary cause of implant failure was likely the laser etching at the fracture interface, though the patient also had risk factors of an elevated BMI and increased implant loading cycles which could contribute to implant failure. Even though revision arthroplasty is a frequent and appropriate occurrence in large volume institutions, it is important to remember that repeat surgery has associated risks, many of which are significantly increased compared to the index procedure. Our patient ultimately had a technically successful revision surgery. However, due to the immobilization in the perioperative period, and despite appropriate deep venous thrombosis prophylaxis, the patient developed a massive pulmonary embolism at the postoperative rehabilitation facility, unfortunately resulting in death.
